# Miglustat therapy in the French cohort of paediatric patients with Niemann-Pick disease type C

**DOI:** 10.1186/1750-1172-7-36

**Published:** 2012-06-07

**Authors:** Bénédicte Héron, Vassili Valayannopoulos, Julien Baruteau, Brigitte Chabrol, Hélène Ogier, Philippe Latour, Dries Dobbelaere, Didier Eyer, François Labarthe, Hélène Maurey, Jean-Marie Cuisset, Thierry Billette de Villemeur, Frédéric Sedel, Marie T Vanier

**Affiliations:** 1 Centre Référence des Maladies Lysosomales, Neuropédiatrie, CHU Trousseau, APHP, 75 571, Paris Cedex, 12, France; 2 Committee for the Evaluation of Treatment for Niemann-Pick diseases (CETNP), Paris, France; 3 Centre Référence des Maladies Héréditaires du Métabolisme de l’Enfant et de l’Adulte (MaMEA), Necker-Enfants Malades, APHP, Paris, France; 4 Maladies Métaboliques, Hôpital des Enfants, CHU, Toulouse, France; 5 Centre Référence des Maladies Héréditaires du Métabolisme, CHU La Timone, APHM, Marseille, France; 6 Centre Référence des Maladies Héréditaires du Métabolisme, CHU Robert Debré, APHP, Paris, France; 7 Laboratoire Gillet-Mérieux, CBPE, Hospices Civils de Lyon, Lyon, France; 8 Centre Référence des Maladies Héréditaires du Métabolisme CHRU, Lille, France; 9 Pédiatrie, CHU, Hautepierre, Strasbourg, France; 10 Médecine Pédiatrique & INSERM U921, CHRU de Tours, Université François Rabelais, Tours, France; 11 Neuropédiatrie, CHU, Kremlin Bicêtre, APHP, France; 12 Neurologie Pédiatrique, CHRU, Lille, France; 13 Université Pierre et Marie Curie, Paris, VI, France; 14 Fédération des Maladies du Système Nerveux, Salpêtrière, APHP, Paris, France; 15 Inserm Unité 820, Université Lyon-1, Lyon, France

**Keywords:** Niemann-Pick disease type C, Paediatric, Miglustat

## Abstract

****Background**:**

Niemann-Pick disease type C (NP-C) is a rare neurovisceral lysosomal lipid storage disease characterized by progressive neurological deterioration. Published data on the use of miglustat in paediatric patients in clinical practice settings are limited. We report findings from a prospective open-label study in the French paediatric NP-C cohort.

****Methods**:**

Data on all paediatric NP-C patients treated with miglustat in France between October 2006 and December 2010 were compiled. All patients had a confirmed diagnosis of NP-C, and received miglustat therapy according to manufacturer’s recommendations. Pre-treatment and follow-up assessments were conducted according to a standardized protocol.

****Results**:**

Twenty children were enrolled; 19 had *NPC1* gene mutations and 1 had *NPC2* gene mutations. The median age at diagnosis was 1.5 years, and the median age at miglustat initiation was 6.0 years. Eight NPC1 patients had the early-infantile, eight had the late-infantile, and three had the juvenile-onset forms of NP-C. A history of hepatosplenomegaly and/or other cholestatic symptoms was recorded in all 8 early-infantile onset patients, 3/8 late-infantile patients, and 1/3 juvenile onset patients. Brain imaging indicated white matter abnormalities in most patients. The median (range) duration of miglustat therapy was 1.3 (0.6–2.3) years in early-infantile, 1.0 (0.8–5.0) year in late-infantile, and 1.0 (0.6–2.5) year in juvenile onset patients. NP-C disability scale scores indicated either stabilization or improvement of neurological manifestations in 1/8, 6/8, and 1/3 NPC1 patients in these subgroups, respectively. There were no correlations between brain imaging findings and disease course. Mild-to-moderate gastrointestinal disturbances were frequent during the first 3 months of miglustat therapy, but were easily managed with dietary modifications and/or anti-propulsive medication.

****Conclusions**:**

Miglustat can improve or stabilize neurological manifestations in paediatric patients with the late-infantile and juvenile-onset forms of NP-C. Among early-infantile onset patients, a shorter delay between neurological disease onset and miglustat initiation was associated with an initial better therapeutic outcome in one patient, but miglustat did not seem to modify overall disease course in this subgroup. More experience is required with long-term miglustat therapy in early-infantile onset patients treated from the very beginning of neurological manifestations.

## **Introduction**

Niemann-Pick disease type C (NP-C) is a rare lysosomal lipid storage disease characterized by neurological deterioration [1,2] with constant progression over time [3-5]. NP-C is caused by autosomal recessive mutations in either one of the two genes, *NPC1* or *NPC2,* which encode proteins involved in the regulation of normal intracellular lipid trafficking [1,6]. It is estimated to affect 1 case in every 100,000–120,000 live births [1,7].

In very rare cases of the severe perinatal (systemic) form of NP-C, patients typically die from liver failure within the first months of life [8,9]. However, NP-C most frequently presents during middle-to-late childhood, and an increasing number of cases are being detected among adolescents and adults [10]. The symptomatology and rate of disease progression of NP-C are strongly influenced by age at onset of neurological manifestations, and different clinical forms have been described on this basis [3,11]. The early-infantile form arises at <2 years of age, the late-infantile form at 2 to 5 years, the juvenile form at 6 to <15 years, and the adolescent/adult form at ≥15 years [2,6,11-13].

Paediatric forms of NP-C tend to feature initial hepatosplenomegaly; an episode of neonatal cholestatic icterus may have occurred [1,8,14]. Later on, neurological manifestations begin to overshadow systemic symptoms. Early delay in motor milestones is often seen in the early-infantile form. Signs of vertical supranuclear gaze palsy (VSGP) are frequent early neurological manifestations, but frequently go undetected until later. Patients may present with clumsiness and progressive cerebellar ataxia. Over time, progressive dysmetria, dystonia, pyramidal signs, dysphagia, dysarthria, cataplexy and/or epileptic seizures, and cognitive impairment often develop [1,2,13,15]. Typically, patients with early-onset neurological manifestations experience more rapid decline and a lower life expectancy than those with later-onset manifestations [1,2].

Miglustat was approved in Europe for the treatment of progressive neurological manifestations in adult patients and paediatric patients with NP-C in January 2009, and has subsequently been approved in a number of other countries, based on data from preclinical studies [16] and clinical trials showing that it can stabilize neurological disease and/or slow its progression [17-21]. Data from a retrospective observational study of miglustat efficacy in a large cohort of NP-C patients aged between 0 and 32 years demonstrated greater beneficial effects of miglustat on neurological disease in adolescents and adults than those seen in children with the earliest forms of NP-C [4].

Data on the therapeutic effects of miglustat in paediatric patients in clinical practice settings are relatively limited [22-27], and evidence from patients with the early-infantile form are particularly scarce. There is therefore an ongoing need for further clinical experience data on the use of miglustat in children, particularly with regard to disease-specific disability assessments. In addition, there are few data on the response of specific neurological manifestations such as epileptic seizures and cataplexy to miglustat therapy.

We report data from a prospective open-label cohort study evaluating disease progression and response to miglustat therapy among all treated paediatric patients with NP-C diagnosed in French hospitals. Findings based on NP-C disability scale assessments, brain imaging and other follow-up assessments conducted according to international disease management recommendations are presented [2].

## **Methods**

### **Patients**

Data on all paediatric NP-C patients treated in France with miglustat between October 2006 and December 2010 were compiled from a network of treatment centres co-ordinated by the French Committee for the Evaluation of Treatment for Niemann-Pick diseases (CETNP). Participating sites included six reference centres and three competence centres.

All index patients had a confirmed diagnosis of NP-C based on filipin staining and molecular genetic laboratory tests. Genetic analyses comprised exon and junction sequencing of the *NPC1* and *NPC2* genes, and in specific cases cDNA sequencing or multiplex ligation-dependent probe amplification (MLPA). Cases with a sibling history of proven NP-C were diagnosed by genetic analysis alone.

### **Treatment**

All patients received miglustat therapy according to manufacturer’s recommendations based on body surface area (BSA) [20]. Doses were escalated up to full doses as per BSA over a period of 3 weeks to 3 months, based on tolerability.

Patients undertook a diet incorporating reduced disaccharide content (decreased saccharose and other carbohydrates) at or after the start of therapy, as advised by investigators according to clinical need. Patients who experienced gastrointestinal disturbances received temporary reductions in miglustat doses and/or symptomatic therapy.

### **Assessments**

Patient pre-treatment and follow-up assessments were conducted according to a standardized protocol that was in accordance with defined international guidelines for disease monitoring in NP-C (Table 1) [2].

**Table 1:** Standard assessments at treatment start and during follow up

**Assessment**	**Treatment start**	**Frequency**
**General**		
· Complete physical examination	**✔**	Every 3–6 months
**Clinical parameters of neurological disease**		
· NP-C functional disability scale	**✔**	Every 6–12 months
· Video recording	**✔**	Every 6–12 months
· Epileptic seizures	**✔**	Every 6–12 months
· Narcolepsy/cataplexy	**✔**	Every 6–12 months
**Other measures**		
· Psychometric evaluations	**✔**	Every 6–12 months
· Hearing	**✔**	Every 6–12 months
· Abdominal ultrasound	**✔**	Depending on initial findings
· Chest X-ray	**✔**	Initial assessment and depending on clinical evolution
**Laboratory parameters**		
· Liver function	**✔**	Every 6–12 months
· Haematology (blood counts)	**✔**	Every 6–12 months
· Plasma chitotriosidase (optional)	**✔**	Initial assessment
**Cerebral imaging**		
· MRI or MRS (magnetic resonance spectroscopy)*	**✔**	Every 12 months

*Determination of Cho/NAA ratio optional.

Pre-treatment assessments included medical histories of systemic and neurological manifestations, clinical examinations, abdominal ultrasound, chest X-ray, electromyography, and laboratory tests (including haematology, liver markers and optional plasma chitotriosidase activities). Specific examinations were video-recorded.

Regular assessments of seizures (with electro-encephalography [EEG]) and cataplexy were conducted, when required. Other evaluations for characteristic NP-C manifestations included psychometric testing of neuropsychological impairment, ophthalmological examination including saccadic eye movement (SEM) abnormalities, and changes in hearing based on brainstem auditory evoked potentials (BAEP).

Cerebral imaging was conducted based on magnetic resonance imaging (MRI) [28], which included T1, FLAIR and T2-weighted sequences. When possible, magnetic resonance spectroscopy (MRS) was also conducted (often under general anaesthesia) with a single voxel at long (TR1500 ms/TE 135 or 144 ms) and short (TE 28 ms) echo times in the centrum semi-ovale. The surfaces of metabolite peaks (N-acetyl-aspartate [NAA], creatine [Cr] and choline [Cho]) were integrated, and the NAA/Cr, Cho/Cr and Cho/NAA ratios were calculated and compared with normal values in age-matched children.

Patient scores on a published NP-C specific disability scale [11], which assesses four key parameters of neurological disease progression (ambulation, manipulation, language, swallowing) were measured before treatment and at multiple time points during follow up. A modified version of the original scale was used, which assigns scores from 0 (best) to 1 unit (worst), with equal weighting for each parameter [4].

No statistical analyses were performed as this was an open-label study with no pre-defined hypotheses. Descriptive statistics were used to describe observed clinical changes. All results are stratified according to established forms of neurological disease in NP-C, based on age at neurological disease onset [2].

## **Results**

### **Patients**

A total of 20 children born between 1994 and 2010 were included in the study (11 females and 9 males), among whom 19 had mutations in the *NPC1* gene and 1 had mutations in the *NPC2* gene. Cases 12 and 13 are siblings.

All patients were fully genotyped (Table 2); in 17/19 families the genotypes of the patients’ parents were also characterised to establish segregation of alleles.

**Table 2:** Systemic and neurological symptoms before miglustat therapy

		**Gender**	**Visceral**			**Neurological**							
**Patient**	**NP-C gene mutations**		**Cholestasis**	**HSM**	**Lung involvement** **(specific)**	Motor or cognitive deficits*	**Cerebellar ataxia (clumsiness)**	**Dysarthria (speech delay)**	**Dystonia**^†^ **(distal motor deficit)**	**Dysphagia**	**VSGP**	**Cataplexy**	**Epilepsy**
**Perinatal visceral**													
#1	*NPC2* C99R/C99R	F	Yes (and cirrhosis)	Yes	No	NA	NA	No	No	Yes	No	No	No
**Early-infantile**													
#2	L830P/R958X	F	Yes	Yes	No	Yes	No	No	(Yes)	Yes	Yes	No	No
#3	C63fsX75/C63fsX75	F	Yes	Yes	No	Yes	No	No	(Yes)	No	Yes	No	No
#4	T1205R/T1205K	M	No	Yes	Yes	Yes	No	(Yes)	(Yes)	No	Yes	No	No
#5	IVS21-2delATGC/ IVS21-2delATGC	M	Yes	Yes	Yes	Yes	No	No	No	No	No	No	No
#6	G1195V/G1195V	M	Yes	Yes	No	Yes	No	(Yes)	No	No	No	No	No
#7	P543L/IVS14 + 1G > A	F	Yes (and cirrhosis)	Yes	Yes	Yes	No	(Yes)	No	No	Yes	No	No
#8	P543L/T1205fs	F	Yes	Yes	Yes	Yes	No	(Yes)	No	Yes	No	No	No
#9	T1036M/T1036M	F	Yes (foetal)	Yes	Yes	Yes	No	(Yes)	(Yes)	No	No	No	No
**Late-infantile**													
#10	Y509C/del exon4	F	No	Yes	No	Yes	(Yes)	(Yes)	No	Yes	Yes	No	No
#11	I1061T/D242H	M	Yes	Yes	No	Yes	Yes	Yes	No	Yes	Yes	Yes	No
#12	P1007A/T1205K	M	No	No	No	Yes	Yes	No	No	Yes	Yes	Yes	Yes
#13	P1007A/T1205K	F	No	No	No	Yes	Yes	No	No	No	Yes	Yes	Yes
#14	R518W/G992W	M	No	No	No	Yes	(Yes)	(Yes)	No	No	Yes	No	No
#15	A470P + I837V/A470P + I837V	M	No	Yes	No	Yes	Yes	No	No	No	Yes	No	No
#16	R518W/D944N	F	No	No	No	Yes	Yes	Yes	No	No	Yes	No	Yes
#17	I1061T/R934X	M	No	No	No	Yes	Yes	Yes	No	Yes	Yes	No	Yes
**Juvenile**													
#18	I1061T/Q421X	M	No	Yes	No	Yes	Yes	No	No	No	Yes	No	No
#19	Q991fsX1005/W1143R	F	No	No	No	Yes	No	No	No	Yes	Yes	Yes	Yes
#20	I1061T/I1061T	F	No	No	No	Yes	Yes	Yes	Yes	Yes	Yes	No	Yes

*Motor deficits (hypotonia and motor delay) in early-infantile onset patients, cognitive deficits (learning difficulties) in late-infantile and juvenile onset patients;^*†*^*distal motor deficit in early-infantile patients due to peripheral neuropathy, and ‘dystonia’ in late-infantile and juvenile patients. HSM = hepatosplenomegaly; VSGP = vertical supranuclear gaze palsy.*

Among the NPC1 patients, eight were classified as having the early-infantile form, eight as having the late-infantile form, and three as the juvenile form, as defined by the age of neurological onset [12]. Overall, the median (range) age at neurological disease onset was 3.0 years (5 months to 7 years). The median (range) age at diagnosis was 1.5 years (prenatal to 14 years), and the median (range) age at start of miglustat therapy was 6.0 years (2 months to 14.8 years).

### **Pre-treatment disease history**

#### ***Patient with perinatal visceral disease***

Patient 1 developed cholestasis and hepatosplenomegaly at 1 month of age. This patient was homozygous for the well described *NPC2* mutation, p.C99R, which has previously been associated with either a perinatal lethal disease or an early-infantile neurological form in the same sibship [29,30]. No disability scale or brain imaging assessments were performed in this child, but clinical examination revealed hypotonia, due either to the severity of his liver disease or to early cerebral disease. Haematopoietic stem cell transplantation was declined due to his poor general condition.

#### ***Patients with early-infantile neurological onset***

All eight patients with early-infantile neurological disease onset had a history of hepatosplenomegaly, and all but one had prolonged neonatal cholestasis; liver biopsy revealed evidence of cirrhosis in two patients (Table 2). One patient (#7) had severe portal hypertension with oesophageal varices (but no digestive bleeding) at 5 months of age. Five patients had a history of pulmonary involvement, but only two (#4 and #5) had specific alveolar or interstitial pulmonary disease detected by chest X-ray at 6 months of age and confirmed by bronchoalveolar lavage revealing accumulation of foamy macrophages: patient 4 needed oxygen therapy up to 15 months of age, and currently has frequent pulmonary infections; patient 5 had frequent bronchitis with interstitial signs on chest X-ray around 6 months of age and then developed alveolar proteinosis with progressive respiratory failure. Patient 6 had repeated pulmonary infections from birth to 3 months of age. Patient 8 had sub-acute aspiration pneumonia at 2 years of age without specific biological signs on bronchopulmonary lavage. Patient 9 had bronchopulmonary dysplasia presumably due to prematurity, and which required non-invasive ventilation up to 6 months of age.

Neurological manifestations among early-infantile patients appeared between 5 and 12 months of age, and included initial hypotonia, delayed motor development and swallowing disorders. VSGP was observed at 9, 18, and 24 months of age in five patients, but no patients had cataplexy. One patient had pronounced dysphagia and subsequent feeding difficulties at 5 months of age, requiring enteral feeding with nasogastric tube followed by gastrostomy aged 9 months. Four patients exhibited clinical signs of peripheral neuropathy, which included distal motor deficit, dysesthesia and diminished osteotendinous reflexes. In each case a myelinic neuropathy was confirmed by electrodiagnostic testing.

Filipin staining, performed in fibroblasts from each patient, invariably showed a massive accumulation of unesterified cholesterol in perinuclear vesicles (classic phenotype). The *NPC1* mutant allele p.I1061T was not observed in this age subgroup, in good accordance with our previous observations [6,30,31]. The p.P543L mutation (present in patients 7 and 8) has previously been reported to lead to an early-infantile form of NP-C [29]. Mutations p.G1195V and p.L830P were detected in one patient each; to our knowledge these mutations have not previously been reported.

#### ***Patients with late-infantile neurological onset***

Three of the eight late-infantile onset patients had splenomegaly, among whom only one also had a history of neonatal cholestasis (#11 – his elder brother died from foetal hydrops due to NP-C). No patients in this subgroup exhibited pulmonary disease. Neurological manifestations appeared between 2 and 5 years of age, and included initial slow motor function and clumsiness or ataxia, delayed language development, and behavioural disturbances with relational troubles. All patients exhibited VSGP, and 5/8 patients had cataplexy and/or epilepsy. Because of pronounced dysphagia and related feeding difficulties, gastrostomy tube and discontinuous enteral feeding became mandatory before initiation of miglustat in patient 12 (at 12 years of age) and patient 17 (at 10 years of age). Another patient (#14) underwent gastrostomy a few weeks after miglustat initiation because he refused oral treatment due to its bitter taste.

A ‘classic’ filipin staining result was observed in fibroblasts from all cases except patient 16, including patient 12 who had one p.P1007A allele, which is usually associated with a ‘variant’ filipin staining pattern [31]. The p.1061 T mutation constituted 2/14 of the mutant alleles among 7 unrelated patients in this age subgroup. Mutations detected in patients 10 and 15 have not previously been described. Two patients (#14 and #16) were heterozygous for the p.R518W mutation, which in the homozygous state has previously been reported in adult-onset patients [10].

#### ***Patients with juvenile neurological onset***

Only one juvenile-onset patient had visceral symptoms: enlarged spleen without a history of neonatal cholestasis. No patients in this subgroup exhibited pulmonary disease. Neurological manifestations started between 5 and 7 years of age in all three juvenile-onset patients, and included praxis disorders, a cerebello-dystonic syndrome, cognitive decline and swallowing disorders (but no psychiatric signs). All patients had VSGP. Two patients (#’s 19 and 20) experienced epileptic seizures and cataplectic episodes before miglustat therapy, at the ages of 9 years and 12 years, respectively. At the age of 5 years, one patient (#18) diagnosed earlier based on visceral symptoms was found to have deafness, a probable early sign of the disease.

As regards biochemical phenotype and genotype, a ‘classic’ filipin pattern was found in all juvenile-onset patients. The p.1061 T mutation constituted 3/6 of the mutant alleles detected in this age subgroup.

### **Miglustat therapy and effects on neurological disease**

**Figure 1:**
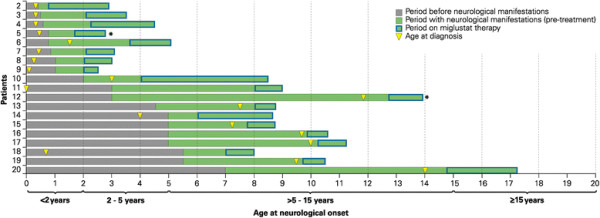
**Key age milestones for NPC1 patients.** Each horizontal bar represents age milestones for an individual patient. Data included only for NPC1 patient; *Patient 5 died aged 2 years and 9 months, and patient 12 died aged 13 years 11 months.

Key age milestones (periods before and with neurological manifestations, age at diagnosis and duration of miglustat therapy) are summarized in Figure 1. Details of miglustat therapy and changes in neurological disease status are summarized in Table 3.

**Table 3:** Miglustat therapy and neurological evolution during follow up

**Patient**	**Age at onset of neurological manifestations**	**Age at start of miglustat therapy**	**Miglustat dose (mg/day)**	**Duration of miglustat therapy**	Disease evolution*
**Perinatal visceral**					
#1	2 months	2 months	50	2 months	NA^*†*^
**Early infantile**					
#2	5 months	9 months	100–150-100	22 months	Initially improved then worsened
#3	6 months	2 years 1 month	200	18 months	Worsened
#4	7 months	2 years 3 months	150–300–150	27 months	Worsened
#5	9 months	20 months	200	13 months	Worsened^*†*^
#6	9 months	3 years 7 months	200–300–150	18 months	Stabilized
#7	10 months	2 years 2 months	200–150–200	12 months	Worsened
#8	12 months	2 years	50–100	12 months	Worsened
#9	12 months	2 years	100–200	8 months	Worsened
**Late infantile**					
#10	2 years	4 years	250	60 months	Stabilized
#11	3 years	8 years	100–300	12 months	Improved
#12	3 years	12 years 7 months	600–400	12 months	Worsened^*†*^
#13	4 years 6 months	8 years	400	9 months	Worsened^Ŧ^
#14	5 years	6 years	150–250	36 months	Worsened transiently then stabilized
#15	5 years	7 years 9 months	200–300	12 months	Stabilized^Ŧ^
#16	5 years	9 years 10 months	400–200–400	9 months	Worsened transiently then improved
#17	5 years	10 years 3 months	300	12 months	Improved
**Juvenile**					
#18	5–6 years	7 years	300	12 months	Worsened^Ŧ^
#19	5–6 years	9 years 9 months	400	7 months	Worsened then stable
#20	6–8 years	14 years 9 months	600	30 months	Improved then stable

^*†*^Patient 1 died aged 4 months, patient 5 died aged 2 years and 9 months, and patient 12 died aged 13 years 11 months; *disease evolution based on NP-C disability scale[11]*and global clinical judgment; NA: not applicable due to young age*^*Ŧ*^*miglustat treatment was stopped in patient #’s 13, 15 and 18 after 9, 12 and 12 months, respectively.*

#### ***Patients with early-infantile neurological onset***

The median (range) interval between the onset of neurological manifestations and initiation of miglustat therapy was 14 (4–34) months, and the median (range) age at miglustat start was 25 (9–43) months. Early-infantile patients received miglustat for a median (range) of 16 (8–27) months.

**Figure 2:**
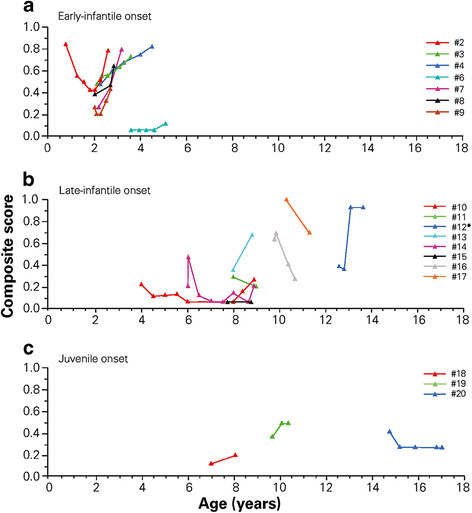
**Changes over time in individual patient composite scores on the NP-C disability scale during miglustat treatment.** Patients with **a**) early-infantile, **b**) late-infantile and **c**) juvenile-onset disease; data included for NPC1 patients only; no disability scale data available from early-infantile patient 5, who died aged 2 years and 9 months; *patient 12 died at 13 years 11 months of age.

Based on disability scale assessments, key neurological parameters were stabilized in 1/8 (13%) patients (Figure 2a). Patient 6, who started miglustat aged 3.6 years, was essentially stabilized throughout 18 months on miglustat. In spite of showing initial neurological signs at 9 months of age, the evolution of disease before miglustat therapy in this patient was similar to that seen in the late-infantile onset form (i.e. a slower disease progression). Patient 2, who had the earliest neurologic onset in this group (at 5 months), started miglustat at the age of 9 months and showed consistent improvement throughout 15 months of therapy, but began to deteriorate after Month 18. By Month 22 of therapy her disability score was similar to that at initiation of miglustat.

During a mean treatment period of approximately 18 months, disability scale scores increased (indicating disease progression) in five patients in spite of slight improvements in interactions, tonus and/or salivation. All but one exhibited pyramidal tract involvement. Three patients required discontinuous enteral feeding by nasogastric tube (#7, persistence of severe portal hypertension with oesophageal varices contra-indicated the gastrostomy) or by gastrostomy tube (#’s 3 and 8) after 10 to 12 months of miglustat therapy, and one patient (#4) developed epilepsy at the age of 32 months. NP-C disability scale data were not obtained for patient 5, whose steady respiratory worsening was associated with neurological disease progression during a 13-month period of therapy; the severity of his respiratory condition precluded disability scale assessments.

#### ***Patients with late-infantile neurological onset***

The median (range) interval between the onset of neurological manifestations and initiation of miglustat therapy was 4.2 (1.0–9.6) years, and the median (range) age at miglustat start was 8.0 (4.0–12.6) years. The median (range) treatment duration at last evaluation was 1.0 (0.8–5.0) years.

Disability scale scores indicated improvement/stabilization of neurological parameters in 6/8 (75%) patients in this subgroup (Figure 2b). Patients 11 and 17 both improved during 12 months of miglustat therapy. Patient 17, whose epilepsy was very active before miglustat, showed a global improvement and became free of seizures after months 5 on miglustat. Patient 16 showed initial deterioration during the first month of treatment, and improved later on. Patient 15 showed stabilization during 12 months on miglustat before treatment was stopped because of adverse events. Two patients (#’s 10 and 14) showed overall improvement, but epileptic and cataplectic episodes started 44 months after and just after miglustat initiation, respectively. In patient 14, seizures and cataplectic episodes are controlled using symptomatic medications. In patient 10, neurological deterioration began when epilepsy became medication-resistant (persistence of one to three short tonic seizures per day despite antiepileptic polytherapy). Patient 12 exhibited pronounced worsening during the initial 6 months of treatment, but appeared stabilized (albeit at a high disability score) after 12 months. Patient 13 experienced difficulties to ingest miglustat powder due to its bitter taste and worsened during therapy, showing more active epilepsy: miglustat was discontinued after 9 months.

#### ***Patients with juvenile neurological onset***

The median (range) interval between the onset of neurological manifestations and initiation of miglustat therapy was 4.3 (1.5–7.75) years, and the median (range) age at miglustat start was 9.8 (7.0–14.8) years. The median (range) treatment duration at last evaluation was 1.0 (0.6–2.5) years.

Disability scale scores indicated improvement of neurological manifestations in one of the three patients (Figure 2c). Patient 20 showed initial improvement then stabilized. Cataplectic episodes began in this patient at Month 22; her cataplectic episodes and epilepsy are currently controlled using symptomatic therapy. Patient 19 worsened during the first 3 months of miglustat therapy and then appeared stable between 4 and 7 months of treatment, showing less dystonia but more swallowing difficulties. This patient’s epilepsy became more active during miglustat treatment but was stabilized following alteration of antiepileptic therapy. Patient 18 displayed worsening of neurological manifestations during 12 months of miglustat therapy before treatment was stopped.

### **Electrophysiological findings**

#### ***Patients with early-infantile neurological onset***

Slight non-specific abnormalities such as bioccipital slow waves or slow background activity were identified in EEG analyses before and during miglustat therapy. Rare posterior spikes were observed in patient 4 when he developed epilepsy aged 32 months, after 5 months on miglustat therapy.

Hearing was normal in all patients in this subgroup, although BAEP showed prolonged latencies in two patients aged 2 and 3 years.

Pre-existing electromyographic signs of myelinic neuropathy in four patients did not worsen after 12–24 months in four patients. Clinical signs of myelinic neuropathy worsened in patient 2 after 22 months on miglustat, which correlated with viral infection and simultaneous losses in the ability to stand and language, as well as complete dysphagia. This worsening neuropathy is considered as being related to disease progression.

#### ***Patients with late-infantile neurological onset***

Several non-specific signs were observed in EEG assessments. EEG findings were normal or showed slow waves or a slow background activity in two patients without epilepsy (#’s 11 and 15), and in two patients (#’s 10 and 14) before epilepsy. Various types of electro-clinic seizures were observed before miglustat start (patients 12, 13, 16 and 17), or during miglustat treatment (patients 10 and 14), including atypical or myoclonic absence, generalized tonic-clonic, and focal seizures. After the start of epilepsy, EEG abnormalities were more active, showing focal, multifocal or generalized interictal spikes or spike-waves.

Late-infantile onset patients did not develop hearing impairment, although BAEP showed prolonged latencies in four patients aged between 8 and 9.5 years (#’s 11, 13, 14 and 16). Findings were normal in the three other patients.

#### ***Patients with juvenile neurological onset***

Juvenile-onset patients exhibited a similar profile of EEG abnormalities as that seen in patients with late-infantile onset. For one patient (#18), early-onset deafness at the age of 5 years was considered a sign of neurological disease onset, and required a hearing prosthesis. No hearing loss was observed in the other two juvenile patients.

### **Imaging findings**

Findings from MRI and MRS assessments before therapy and at follow up are summarized in Table 4.

**Table 4:** Brain imaging findings

**Patient**	**MRI before miglustat**	**Follow-up MRI on miglustat**	**MRS before miglustat**	**MRS follow-up on miglustat**
**Early infantile**				
#2	Slight white matter atrophy	M9: stable	ND	M6: normal
#3	Deep WMSA	M12: white matter atrophy affecting the corpus callosum and cortex	Normal	ND
#4	Deep WMSA and cortical atrophy	M24: severe WMSA	Low NAA and high Cho	M12–M24: low NAA, normal Cho, high myo-inositol
#5	ND	ND	ND	
#6	Deep WMSA and minimal atrophy of the cerebellar vermis	M12: stable	Normal	M12: normal
#7	Periventricular and oval centre WMSA	M12: thinning of the periventricular white matter	Normal	M12: low NAA, high Cho/NAA ratio
#8	Periventricular and subcortical WMSA, and thinning of the corpus callosum and middle cerebellar peduncles	M12: stable	ND	M12: low NAA
#9	Diffuse WMSA	M8: diffuse WMSA and slight atrophy	Low NAA, high Cho, high Cho/NAA	M8: stable
**Late infantile**				
#10	Periventricular WMSA	M12–M 48: slowly progressing white matter atrophy	Normal	M12–M48: low NAA, normal Cho
		M54: stable		
#11	Normal	ND	ND	ND
#12	Diffuse WMSA	M12: overall atrophy especially in the cerebellar vermis	Normal	ND
#13	Diffuse WMSA	M9: slight cerebellar atrophy	Normal	ND
#14	Normal	M18: signs of cortical-subcortical atrophy in the sub- and sus-tentorial regions	Normal	M18: low NAA, high Cho, M24: normal
#15	WMSA in the left oval centre, and onset of cerebellar atrophy	ND	NAA/Cr ratio reduced	ND
#16	Periventricular WMSA, cortical and cerebellar atrophy	ND	Normal	ND
#17	Normal	ND	ND	ND
**Juvenile**				
#18	Periventricular WMSA	M12: stable	ND	M12: normal
#19	Posterior periventricular WMSA and slight cortical atrophy	ND	Normal	ND
#20	Normal	M18 and M30: slight cortical atrophy	M0: high myo-inositol	M18: high myo-inositol, M30: high Cho/NAA ratio

Cho = choline; M = month on miglustat therapy; MRI = magnetic resonance imaging; MRS = magnetic resonance spectroscopy; NAA = N-acetylaspartate; ND = not done; WMSA = white matter signal abnormalities.

#### ***Patients with early-infantile neurological onset***

The seven living early-infantile patients showed white-matter abnormalities indicative of delayed myelination or demyelination before miglustat therapy, with three showing atrophy in the periventricular or subcortical regions or the corpus callosum, and two also showing slight atrophy of the cerebellar vermis or peduncles (Table 4). Patient 9 had a normal MRI at 5 months of age before developmental delay was noted at 12 months. These white matter abnormalities remained stable in patients 8 and 9, but worsened slightly during miglustat treatment in the three other patients (#’s 3, 4 and 7), who experienced clinical worsening. The two patients who showed initial improvement (#2) or clinical stabilization (#6) had stable MRI abnormalities and normal MRS at pre-treatment assessment and at 12-month follow up. Patient 3, who showed clinical worsening, also had normal MRS findings at pre-treatment.

Pre-treatment MRS showed low NAA and high Cho peaks in 2/5 patients (#’s 4 and 9). Low NAA peaks were noted at Months 8–24 of miglustat therapy in four patients (#’s 4, 7, 8 and 9) who showed clinical worsening, with normal Cho peaks in patients 4 and 8.

#### ***Patients with late-infantile neurological onset***

Before starting miglustat, three late-infantile onset patients (#’s 11, 14 and 17) had normal brain MRI findings at 6, 8 and 10 years of age, respectively. Five patients had slight periventricular or more generalized white matter abnormalities on MRI (#’s 10, 12, 13, 15, and 16), with cortical or slight cerebellar atrophy also seen in two patients (#’s 15 and 16).

Four patients developed cortical, subcortical or cerebellar signs of atrophy after the commencement of miglustat therapy, two of whom (#’s 10 and 14) experienced stabilization of neurological symptoms, while patients 12 and 13 worsened. Follow-up MRI findings are not yet available for four patients (#’s 11 and 17 who had normal findings before therapy, and #’s 15 and 16).

MRS findings were normal before treatment in patients 10, 12, 13, 14 and 16, and showed a low NAA peak in patient 15. At follow up, patient 10 showed a persistent low NAA peak. Patient 14 had a transient low NAA peak at Month 18 on miglustat, and MRS findings became normal at Month 24. A high Cho peak was also noted at Month 12 in patient 10 and at Month 18 in patient 14, which contrasted with a slight improvement of symptoms.

#### ***Patients with juvenile neurological onset***

Magnetic resonance imaging analysis revealed slight periventricular white matter abnormalities in patient 18 before therapy and at Month 12 of follow up during miglustat therapy, but MRS findings were normal. Periventricular white matter abnormalities were associated with cortical atrophy in patient 19 before therapy, but no MRI follow-up is yet available for this patient, who has so far received 7 months of miglustat treatment. In patient 20, imaging findings were normal before miglustat at the age of 14 years and 9 months, but follow-up analysis showed slight cortical atrophy after 12 months of miglustat therapy and a high Cho/NAA ratio after 30 months, associated with clinical stabilization.

### **Safety and tolerability**

Fifteen out of 20 patients (75%) in this paediatric cohort experienced adverse effects that were considered related to miglustat therapy, including diarrhoea, abdominal pain, anorexia and weight loss. Gastrointestinal adverse events occurred mostly during the first 3 months of miglustat therapy, and usually resolved during continued therapy at lower doses, following institution of a disaccharide-free diet and/or administration of symptomatic treatment (e.g. loperamide).

Most adverse events were mild or moderate in severity, except in three cases where adverse events led to discontinuation of miglustat therapy. Asthenia and/or persistent diarrhoea motivated a decision to stop miglustat therapy after 1 year in one late-infantile onset patient (#15) and one juvenile-onset patient (#18). These adverse events resolved without clinical sequelae after withdrawal of miglustat.

Patient 13 experienced persistent diarrhoea, but refused the recommended disaccharide-free diet and became anorexic: miglustat was subsequently discontinued after 9 months of therapy. One late-infantile onset patient (#14) refused to ingest oral miglustat powder due to its bitter taste, and a gastrostomy was conducted to enable drug administration. This patient subsequently received a normal oral diet and miglustat was well tolerated. Miglustat commercial 100-mg capsules were repackaged in smaller capsules of 50 mg to allow easier swallowing for patient 7. Patient 9 remains strongly constipated on miglustat treatment in spite of laxative medications. Patient 20 presented at the age of 16 years (1 year after starting miglustat therapy) with rectal fistula that persisted despite many treatments. She was later discovered to have Crohn’s disease, which was subsequently controlled with symptomatic therapy (mesalamine) to allow continued miglustat treatment.

Three patients died during follow up. The patient with perinatal visceral disease (i.e. NPC2; #1) developed cholestasis and hepatosplenomegaly at 1 month of age, and died aged 4 months (after 2 months of miglustat therapy) due to liver failure. One early-infantile onset patient (#5) died aged 2 years and 9 months due to respiratory failure with alveolar proteinosis, which was considered to be associated with neurological disease progression during the patient’s 13-month period of miglustat therapy. One late-infantile onset patient (#12) died aged 13 years and 11 months due to aspiration pneumonia.

## **Discussion**

Assessments of key parameters of neurological disease progression based on the published NP-C disability scale indicated either stabilization or improvement of neurological manifestations in 1/8 early-infantile, 6/8 late-infantile, and 1/3 juvenile-onset NP-C patients who received miglustat in this multicentre, open-label cohort study. Beneficial therapeutic effects were seen more frequently in patients with late-infantile/juvenile neurological disease onset than in those with early-infantile onset.

In agreement with previous reports, visceral disease (prolonged neonatal cholestasis and/or hepatosplenomegaly) was more prevalent among early-infantile onset patients than in later-onset patients in this paediatric cohort [1,26]. Neonatal cholestasis healed in all NPC1 patients before miglustat therapy, but portal hypertension persisted in one patient during miglustat treatment. While visceral disease was not a focus of disease monitoring in this study, miglustat did not appear to have any effect on hepatosplenomegaly (data not shown) or pulmonary disease (patient 5 died from alveolo-interstitial complications after 13 months of miglustat treatment).

While the NPC2 patient had cholestasis and hepatosplenomegaly from 1 month of age (before initiation of miglustat), she developed hepatic failure during miglustat therapy. It is not possible to ascertain whether miglustat might have worsened the liver disease in this patient as a similar course of disease is quite common in NPC2 [29].

In general, our findings appear in line with those from previous clinical trial data [4,18,19] and case reports [23,25,27] on the effects of miglustat on neurological disease manifestations in paediatric NP-C patients. Based on clinical assessments in a 24-month study of miglustat in children aged 4–12 years, Patterson et al. reported stabilization of SEM, an accepted marker of early neurological deterioration in NP-C, in 67% of patients throughout therapy [18]. Ambulation (measured using the standard ambulation index) was stabilized in 80% of patients, and swallowing (patients’ ability to swallow various substances) remained stable in 90% [18]. Based on the same NP-C disability scale as that employed in the current study, a retrospective analysis of miglustat efficacy in 66 NP-C patients aged between 0 and 32 years (mean ± SD, 9.7 ± 7.6 years) showed that ambulation, manipulation, language and swallowing were stabilized or improved in 75% of patients during an average of 18 months of therapy [4]. Stratification of patients according to age indicated that beneficial effects were greater in juvenile, adolescent and adult patients than in those with disease onset before 6 years of age [4].

Pineda et al. reported clinical experience with the use of miglustat in a paediatric cohort of 16 Spanish NP-C patients, comprising five with the early-infantile form, four with the late-infantile form, and seven with the juvenile form [26]. As in the current study, efficacy assessments were based on an NP-C specific disability scale, albeit a modified version that included scores for the presence of epilepsy and ocular movements. Similar to our findings, the Spanish cohort study indicated that patients with the late-infantile and juvenile-onset forms were more likely to show improvements or stabilization of neurological disease during miglustat therapy compared with patients with severe, early-infantile onset [26]. However, Spanish early-infantile onset patients who showed deterioration during miglustat therapy were at an advanced stage of the disease before starting therapy, while those with a better evolution had started therapy at the youngest ages. Overall, the Spanish data suggested that better treatment effects might be expected when treatment was initiated early, before “irreversible neurological damage” [2,26].

In our cohort, there did not appear to be as strong a correlation between patients’ disability scale scores before treatment and subsequent changes during therapy. However, a short interval between neurological disease onset and the start of miglustat therapy and/or young age at treatment start was associated with a better initial therapeutic outcome in one early-infantile onset patient: in patient 2, who initially improved, the delay between neurological disease onset and initiation of miglustat was only 4 months, and miglustat was commenced at 9 months of age. Other early-infantile patients, four of whom worsened, had a mean (range) delay between neurological disease onset and start of treatment of 1.8 (0.9–2.8) years, and a mean (range) age at treatment start of 2.3 (range 1.7–3.6) years. In the late-infantile and juvenile-onset patients the mean (range) delay to therapy was 3.8 (1.0–7.8) years in patients who were stable or improved after treatment, and 4.8 (1.5–9.6) years for those who worsened. These observations appear to support the argument for starting miglustat treatment earlier, as soon as possible after the onset of neurological symptoms and especially in the early-infantile onset patients.

Data from the current cohort are in line with previous data on the high prevalence of epilepsy and cataplexy in late-infantile and juvenile (but not early-infantile) onset patients [1,2]. Published data on the possible therapeutic effect of miglustat on cataplexy and epilepsy are very scarce. Zarowski et al. have previously reported a complete cessation of cataplectic activity in a young male patient with juvenile-onset NP-C [32]. In our study, miglustat did not appear to prevent the occurrence of, or to systematically improve, cataplexy or epilepsy among the small number of patients in the late-infantile and juvenile-onset subgroups. Limited data from the Spanish paediatric cohort study indicated that the onset of epilepsy and its resistance to symptomatic pharmacotherapy may result in worsening of patients’ scores on the NP-C disability scale [26]. In our series, the presence of pre-existing epilepsy, or its onset during miglustat therapy, did not appear to affect neurological outcome when seizures were stabilized using anti-epileptic therapies. Anti-epileptic drugs employed included sodium valproate, lamotrigin and levetiracetam. Carbamazepine, oxcarbazepine and vigabatrin were avoided as they could promote myoclonias. Phenytoin was also not used in order to avoid possible cerebellar adverse effects.

Auditory acuity remained stable in this series, and no patient experienced worsening of electrical peripheral neuropathy during miglustat therapy.

Magnetic resonance imaging showed white matter abnormalities in NPC1 patients with each of the age-at-onset forms. In general, discrete posterior periventricular white matter abnormalities were followed by more diffuse changes resembling delayed myelination or demyelination among these patients. Cortical or subcortical atrophy tended to appear first in the infantile-onset forms (although it was also present in later-onset forms). Cerebellar atrophy was present in relatively few cases (two early-infantile and three late-infantile patients).

Magnetic resonance spectroscopy identified some abnormalities, including low NAA and/or high Cho with high Cho/NAA ratio. At short echo time, a high myo-inositol peak was observed before therapy and at Month 18 of follow-up in patient 4 (worsened) and patient 20 (stabilized). It is known that progressive neurodegenerative diseases are associated with a decrease of the NAA peak, which is considered to be a marker of neuronal viability, and by an increase of the Cho peak, which is considered to be a marker of membrane destruction or gliosis. Nevertheless there was no consistent pattern of change over time or in response to miglustat in our series. A low NAA peak was associated with cerebral atrophy in 8/18 cases but not with clinical worsening in all of these. This contrasts with a previously published case series based on three adult NP-C patients treated with miglustat for 24 months, where mild clinical improvement or stabilization concurrent with sustained decreases in cerebral Cho/NAA ratio were observed [24].

While our imaging findings are of value in that they add to the relatively limited amount of published data from longitudinal imaging studies in paediatric NP-C patients, it is notable that there were no apparent correlations between MRI or MRS findings and clinical disease course during miglustat therapy. It is possible that methodological and data limitations in our cohort preclude a definitive conclusion on the utility of this imaging technique. MRS analyses for French paediatric patients were conducted at several different sites by several technicians, and according to varied analysis protocols. MRS data follow up beyond 24 months were only available for 4/20 patients, which makes it difficult to assess long-term changes. However our findings do not favour the use of high Cho peak or Cho/NAA ratios as objective markers of therapeutic effect in paediatric patients, as has been proposed for adult patients [24].

A possible correlation between the evolution of neurological manifestations (based on changes in NP-C disability scores) and cerebral hypometabolism (measured using positron emission tomography [PET]) was previously reported based on data from Spanish juvenile- and infantile-onset patients treated with miglustat [26]. Cerebral hypometabolism was stabilized when miglustat appeared to slow the progression of neurological symptoms, and progressive hypometabolism correlated with increasing disability scores [26]. Nevertheless, PET is unlikely to be of practical use for routine clinical monitoring due to the limited availability of equipment.

Mild or moderate gastrointestinal disturbances were frequent during miglustat therapy, but usually resolved within the first 3 months of treatment. In addition, gastrointestinal adverse events were easily managed in most cases by the adoption of dietary alterations, by progressive initiation of miglustat treatment, or by the use of symptomatic therapy (e.g. loperamide). In particular, dietary modifications such as reduced consumption of dietary sucrose, maltose and lactose have been shown to improve the gastrointestinal tolerability of miglustat, and to reduce the magnitude of any changes in body weight, particularly if initiated at or before the start of therapy [33,34]. Finally, observed factors that appear to contribute to reduced treatment compliance among the youngest patients include the bitter taste of oral miglustat therapy, and the lack of a paediatric galenic form.

A decision to stop miglustat was taken for two late-infantile patients and one juvenile patient because of persistent adverse events (e.g. asthenia or anorexia) and clinical judgment of insufficient beneficial effects on disease progression. Such choices are made on a case-by-case basis with collaborative discussions between medical staff and parents, as well as detailed consideration of patients’ clinical evolution and quality of life, well in line with the updated recommendations from an expert panel [35].

In spite of the small number of patients and the relatively short period of follow up in the French paediatric NP-C cohort, and in recognition of the invariably progressive course of neurological deterioration in untreated patients [3,5], we conclude that miglustat can improve or stabilize neurological disease progression in paediatric patients with NP-C, particularly those with the late-infantile and juvenile-onset forms. Our data from early-infantile onset patients, who generally exhibit greater symptom severity and more rapid progression of neurological manifestations, indicate that commencement of miglustat at approximately 2 years of age has no sustained global effect on the natural course of the disease. A shorter delay between the onset of neurological manifestations and the start of miglustat therapy was associated with a better initial therapeutic outcome in one early infantile-onset patient in this cohort. However, this patient later exhibited worsening of neurological disease after 2 years of age. More clinical experience in early-infantile onset patients treated at the very beginning of their neurological disease over a longer period is required to more fully assess the therapeutic effects of miglustat in this group.

Current guidelines for the clinical management of NP-C propose that miglustat treatment should be initiated at the onset of neurological signs [2,35]. However, among patients with early-infantile NP-C, the invariable occurrence and high frequency of systemic symptoms particularly neonatal cholestasis and hepatosplenomegaly, often leads to a diagnosis before the appearance of neurologic signs or developmental delay. Regular and thorough clinical examination of these patients, if possible combined with cerebral MRI, could detect neurological problems at their very beginning, leading to earlier initiation of treatment with miglustat.

## **Competing interests**

The authors declare that they have no competing interests.

## **Authors’ contributions**

MTV, FS, TBV, BH, BC and HO conceived the study and participated in its design. BH coordinated the study. BH, MTV and TBV drafted the manuscript. MTV and PL carried out the filipin and molecular genetic studies. All authors participated in collection of data, and have read and approved the final manuscript.

## **Disclosures**

BH has received travel expenses, and been invited to meetings funded and organized by Actelion Pharmaceuticals Ltd., Biomarin, Genzyme Corporation and Shire HGT, and has received presentation honoraria from Actelion Pharmaceuticals Ltd. MTV has received travel expenses, carried out paid and unpaid consultancy work, and presentation honoraria from Actelion Pharmaceuticals Ltd., and has received travel expenses, presentation honoraria, and been invited to meetings funded and organized by Genzyme Corporation and Shire HGT. VV has received travel expenses, research grants and presentation honoraria from Actelion Pharmaceuticals Ltd. FS has received travel expenses, carried out paid consultancy work, and received presentation honoraria from Actelion Pharmaceuticals Ltd. BC has received travel expenses and presentation honoraria from Actelion Pharmaceuticals Ltd. DE, JB and JMC have received travel expenses and presentation honoraria from Actelion Pharmaceuticals Ltd. PL has received presentation honoraria from Actelion Pharmaceuticals France. FL has received travel expenses from Merck Serono and Genzyme Corporation. TBV has received funds for an association (ASEP: for health and progress in paediatrics) from Shire HGT and Genzyme Corporation. DD, HO and HM declare that they have no competing interests.
